# At the Lords' Committee

**Published:** 1890-09-06

**Authors:** 


### At the Lords' Committee.

[By an Eye-Witness.]

The curtain has fallen upon the first act of this drama, and before it rises again there will be plenty of time for reflection. Perhaps this is as well, for at present the first feeling which seems naturally to arise is one of disappointment, tinged with a certain sense of misgiving, lest an enquiry which might be fraught with so much benefit to our hospitals may not become so diffuse as to be of little real value.

The opening sittings of the Committee were quite unsensational, perhaps even a trifle dull. If the witnesses were being called in any order or upon any particular principle, it was not easy to detect what it was. The witnesses themselves were, in some instances, people whose visions, not to say crotchets, are so very well known by all who have the slightest acquaintance with hospital politics that it would not have been difficult to recognise them even if one had entered the Committee-room blindfold. Judging by their attendance, the interest of the public in these early proceedings was not very great: the attendance of three or four reporters for the press was most assiduous, but the general public were conspicuous by their absence, and there was a choice of seats between a rather hard form covered with baize, and the comfortable chairs upholstered in red leather, so much more suggestive of the House of Lords.

But by-and-bye a change came o'er the spirit of the dream. The morning papers reported the evidence more fully, and, under a bold headline, which ran as follows: "The London Hospital—serious evidence."

The aspect of the Committee-room was at once changed: so far from there being a choice of seats, standing room was at a premium, and the atmosphere became heated in more senses than one.

The indictment against the London Hospital was severe, but an unbiassed observer could hardly fail to notice that most damaging statements were made without the slightest sign of pain or reluctance, as if the task in hand was of the nature of a personal triumph rather than the performance of a painful public duty. If a more than usually unpleasant question or comment happened to be made by any member of the Committee smiles would be seen to play over the faces of a certain section of the audience and the remark "delicious" would be overheard, varied by such a phrase as "that is a poke in the eye for so and so," if the speaker chanced to be one of the sterner sex. The existence of a strong prejudice being thus evident it was impossible to resist the reflection that there was a great probability of the judgment being somewhat weak.

Another reflection which arose almost irresistibly to the mind was that if the members of the committee had possessed a little more knowledge of hospital life, a good many questions would not have been asked, and that in consequence a good deal of time would have been saved. For instance, it occupied a long while to thresh out the question whether it was or was not a hardship for a nurse or probationer to have to consult the house physician or house surgeon for some trifling ailment, graver maladies, of course, being always referred to some graver person. Probably anybody acquainted with the subject, would be inclined to reply to the enquiry whether nurses are entirely averse to holding communication with house physicians and house surgeons, by using the word which rose so frequently to the lips of Tweedledee when talking to Alice in Wonderland, namely "contrariwise."

If only the members of the Lords' Committee could occupy the recess by each one of them filling the post say of matron one of the hospitals, how much might be learnt. It might even possibly be found that the nurses' "fourteen hours on duty" is not so very great a hardship after all. Undoubtedly fourteen hours continuous labour is too much for anyone, either man or woman. For an ironer to stand fourteen hours at her ironing board, would be monstrous, and even for the young lady at the post office to spend fourteen hours continuously in selling stamps and receiving telegrams would be exhausting. The closest parallel however to the work of a nurse is the work of a domestic servant, notwithstanding the many and great points of difference. In a well ordered household a domestic servant would certainly be required to be "on duty" at least from seven in the morning until ten at night not necessarily hard at work all the time, but liable always to be called upon to render some service, just as the nurse while on duty is always liable to be called upon for some service to her patients. At present, however, there has been no outcry about the long hours of domestic servants; possibly it may soon come, as there seems to be a general desire nowadays to double everybody's pay and to halve their work, while the demand for the liberty of the subject is so great that the ideal programme seems to be that every one shall do exactly as he pleases and if he will not he shall be made to.

In theory the nurse's fourteen hours on duty may appear to be entirely wrong and nothing more or less than white slavery, but in practise there is room to doubt whether there is any very great hardship in it after all.

Drummond, in his "Natural Law in the Spiritual World," says: "Any principle which secures food to the individual without the expenditure of work is injurious, and accompanied by the degeneration and loss of parts." We commend this as a motto to the C.O.S.

